# Draft and complete genome sequences of 17 *Streptococcus* species

**DOI:** 10.1128/mra.00190-25

**Published:** 2025-08-11

**Authors:** Sam Nooij, Ingrid M. J. G. Sanders, Lesley Schout, Rolf H. A. M. Vossen, Susan L. Kloet, Wiep Klaas Smits, Quinten R. Ducarmon

**Affiliations:** 1 Leiden University Center for Infectious Diseases (LUCID), Leiden University Medical Center 4501, Leiden, the Netherlands; 2 Leiden Genome Technology Center, Leiden University Medical Center 4501, Leiden, the Netherlands; Loyola University Chicago, Chicago, Illinois, USA

**Keywords:** *Streptococcus*, genomics, DNA sequencing, human microbiome

## Abstract

We present 17 near-complete or complete genomes of *Streptococcus* spp. obtained from eight mixed cultures of presumed *Ruminococcus gnavus* isolates. The genomes are classified as eight different *Streptococcus* spp., and three currently have no representative available in databases.

## ANNOUNCEMENT

**Fig 1:**
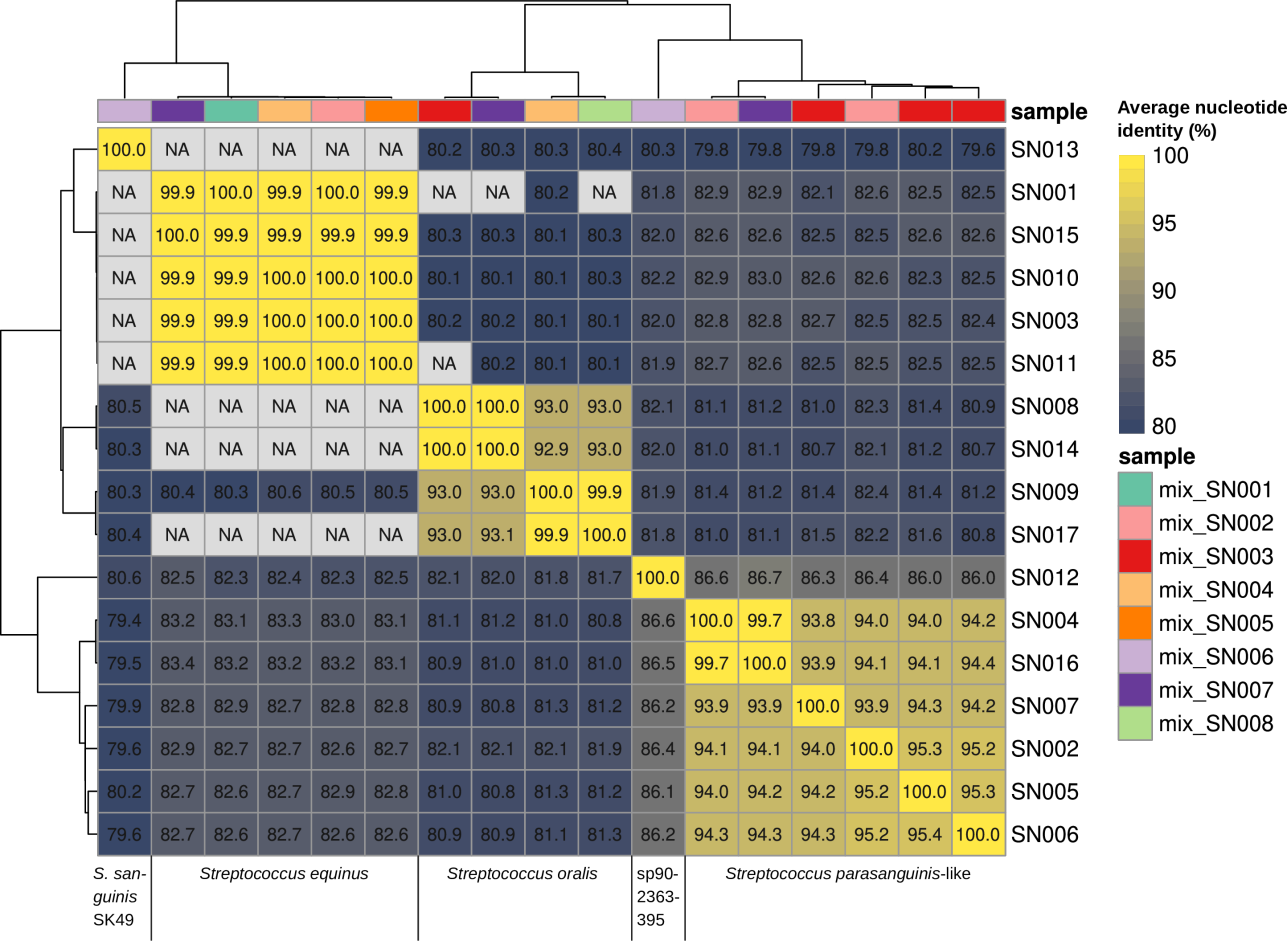
Whole-genome comparison of 17 *Streptococcus* genomes. Whole-genome sequences of 17 *Streptococcus* bacteria were compared using ANI. Values in cells indicate percentage ANI; values much below 80% are reported as NA. Genomes are annotated with the sample they derive from.

We cultured bacteria from healthy human feces and from feces and biopsies of Crohn’s disease patients (1). Bacteria were grown at 37°C on Tryptic Soy Agar with 5% sheep blood (TSS; bioMérieux, Marcy-l'Étoile, France) in an anaerobic cabinet (Whitley A35, Don Whitley Scientific Limited, UK). Selected colonies were confirmed to be *Ruminococcus gnavus* by MALDI-TOF (Bruker Daltonics GmbH, Bremen, Germany) prior to liquid culture in 10 mL brain heart infusion medium (BHI; bioMérieux). DNA was isolated from 5 mL culture using the Qiagen Genomic-tip 100/G (Qiagen, Hilden, Germany) and sheared with the Megaruptor 3 system (Diagenode LLC, Denville, New Jersey, USA) using 35 cycles. Libraries were generated according to “Preparing whole genome and metagenome libraries using SMRTbell prep kit 3.0” (Pacific Biosciences, Inc., California, USA; PN 102-166-600 REV02 MAR2023). Fragments of >5 kb were selected in sub-libraries using diluted AMPure PBbeads (PacBio, 35% beads, 3.1× v/v ratio) or BluePippin (Sage Science, Beverly, Massachusetts, USA), depending on the insert size of the libraries. Libraries were sequenced on the PacBio Sequel IIe platform with a 30 h movie time using Binding Kit 3.2 and Sequencing Kit 2.0. Reads were assembled with Flye (2) (version 2.9.2) without error correction or trimming, and contigs were classified using Contig Annotation Tool (3) (CAT; version 5.2.3) and reoriented to start at *dnaA* for putative chromosomes using dnaapler (4) (version 0.3.0). Eight samples returned (near-)complete *Streptococcus* genomes and short (7–55kbp) predicted *R. gnavus* contigs with 4× estimated depth. We suppose the *Streptococci* are contaminants and cannot determine their exact origin. Nonetheless, we reconstruct and identify these genomes as potentially valuable for the scientific community. We reassembled reads using metaFlye (5) (version 2.9.2), yielding up to 194 contigs per sample (median length: 23 kbp). The assemblies contained one *Streptococcus* contig each, which we separated using seqkit (6) (version 0.16.0). Other contigs were discarded. *Streptococcus* contigs were quality checked by: (i) calculating the length and GC content by QUAST (7) (version 5.0.2), (ii) estimating completeness and contamination with CheckM (8) (version 1.0.13), (iii) annotating genes with Bakta (9) (version 1.6.1), and (iv) classifying their taxonomy with GTDB-Tk (10) (version 2.3.2) using database version r207_v2 (all with default parameters). Relevant statistics are listed in Table 1. We used fastANI (11) (version 1.33) to calculate pairwise average nucleotide identity (ANI; Fig. 1) to determine relatedness between the *Streptococcus* genomes and infer shared sources. We found five *Streptococcus equinus* (NCBI taxonomy; GTDB: *Streptococcus sp001556435*; SN001, SN003, SN010, SN011, and SN015) with highly similar genomes (ANI ≥ 99.9%) from five different samples, four *Streptococcus oralis* (GTDB: 2 × identical *S*. *oralis_S*: SN008, SN014; and 2 × *S. oralis_Y*: SN009, SN017) from three different samples, and six genomes with slightly different classifications that are all close to *Streptococcus parasanguinis* (ANI ≥ 93.8%) from three different samples (1 × 3: SN005, SN006, and SN007; 1 × 2: SN002 and SN004; 1 × 1 genome/sample: SN016). We found one genome classified as *S. sp902363395* (GTDB; SN012), which is unknown to NCBI, that remotely resembles (ANI ~ 86.4%) the *parasanguinis*-like genomes. The final genome was classified as *S. sanguinis* SK49 (GTDB: *S. sanguinis_C*; SN013) and is remotely similar to the other genomes (ANI ≤ 80%).

**TABLE 1:** *Streptococcus* genome assembly characteristics and quality measures^*a*^,^*b*^

Genome	Number of contigs	Length (bp)	Predicted genes	GC%	Circular?	Species	CheckM completeness	CheckM contamination	Assembly method	Depth of coverage	Assembly accession number	Number of reads	Read N50
SN001	1	2,140,162	1,914	40.12	Y	*S. equinus*	99.69	0.15	metaFlye	17	GCA_964272725	21,846	8,750
SN002	1	2,223,664	2,093	41.52	N	*Streptococcus* sp. HMSC072G04	100	0.09	Flye	13	GCA_964272665	40,087	11,112
SN003	1	2,141,365	1,913	40.11	Y	*S. equinus*	99.69	0.15	Flye	21	GCA_964272605	40,087	11,112
SN004	1	2,160,498	2,021	42.06	N	*Streptococcus* sp.	100	0.17	metaFlye	18	GCA_964272625	40,087	11,112
SN005	1	2,125,957	2,016	41.72	Y	*S. parasanguinis*	99.66	0.11	Flye	120	GCA_964272445	34,480	9,258
SN006	1	2,081,198	1,934	42.11	Y	*S. parasanguinis*	100	0.07	Flye	120	GCA_964272375	34,480	9,258
SN007	1	2,118,644	2,017	41.95	Y	*Streptococcus* sp.	100	0.23	Flye	158	GCA_964272575	34,480	9,258
SN008	1	2,068,017	1,940	41.06	Y	*S. oralis*	99.87	0.04	Flye	25	GCA_964272495	34,480	9,258
SN009	1	2,117,116	2,018	40.83	Y	*S. oralis*	99.87	0.06	metaFlye	24	GCA_964272365	17,013	10,226
SN010	1	2,147,719	1,922	40.12	Y	*S. equinus*	99.69	0.15	metaFlye	25	GCA_964272565	17,013	10,226
SN011	1	2,141,236	1,914	40.11	N	*S. equinus*	99.69	0.15	metaFlye	14	GCA_964272615	34,927	10,344
SN012	1	2,025,938	1,881	42.21	Y	*Streptococcus* sp.	100	0.17	metaFlye	21	GCA_964272595	39,626	9,463
SN013	1	2,316,732	2,233	43	N	*S. sanguinis*	100	0	metaFlye	20	GCA_964272475	39,626	9,463
SN014	1	2,068,026	1,939	41.06	Y	*S. oralis*	99.87	0.04	metaFlye	117	GCA_964272555	35,176	10,093
SN015	1	2,141,124	1,914	40.12	Y	*S. equinus*	99.69	0.15	metaFlye	87	GCA_964272695	35,176	10,093
SN016	1	2,166,044	2,028	42.02	Y	*Streptococcus* sp.	100	0.17	Flye	153	GCA_964272405	35,176	10,093
SN017	1	2,119,531	2,017	40.83	Y	*S. oralis*	99.87	0.6	metaFlye	48	GCA_964272485	18,655	9,721

^*a*^ Initial assemblies were made with Flye. In the case of multiple linear contigs, a secondary assembly was made using metaFlye.

^*b*^ The most complete assembly was selected from either approach.

## Data Availability

This project has been deposited in the ENA under accession number PRJEB76410. Separate accession numbers for each assembly are listed in Table 1. Mixed-species reads are available through ERR13354980, ERR13354981, ERR13354982, ERR13354983, ERR13354984, ERR13354985, ERR13354986, ERR13354987, and ERR13354988.
